# Physical Activity Monitoring and Acceptance of a Commercial Activity Tracker in Adult Patients with Haemophilia

**DOI:** 10.3390/ijerph16203851

**Published:** 2019-10-12

**Authors:** Juan J. Carrasco, Sofía Pérez-Alenda, José Casaña, Emilio Soria-Olivas, Santiago Bonanad, Felipe Querol

**Affiliations:** 1 Physiotherapy in Motion, Multispeciality Research Group (PTinMOTION), Department of Physiotherapy, University of Valencia, Carrer de Gascó Oliag, 5, 46010 Valencia, Spain; juan.j.carrasco@uv.es (J.J.C.); felipe.querol@uv.es (F.Q.); 2 Intelligent Data Analysis Laboratory, Department of Electronics Engineering, University of Valencia, Avda. Universitat, 46100 Burjassot, Spain; emilio.soria@uv.es; 3 Haemostasis and Thrombosis Unit, University and Polytechnic Hospital La Fe, Avinguda de Fernando Abril Martorell, 106, 46026 Valencia, Spain; bonanad_san@gva.es; 4 Exercise Intervention for Health Research Group (EXINH-RG), Department of Physiotherapy, University of Valencia, Carrer de Gascó Oliag, 5, 46010 Valencia, Spain; jose.casana@uv.es

**Keywords:** exercise, physical activity, haemophilic arthropathy, fitness tracker

## Abstract

Physical activity (PA) is highly beneficial for people with haemophilia (PWH), however, studies that objectively monitor the PA in this population are scarce. This study aimed to monitor the daily PA and analyse its evolution over time in a cohort of PWH using a commercial activity tracker. In addition, this work analyses the relationship between PA levels, demographics, and joint health status, as well as the acceptance and adherence to the activity tracker. Twenty-six PWH were asked to wear a Fitbit Charge HR for 13 weeks. According to the steps/day in the first week, data were divided into two groups: Active Group (AG; ≥10,000 steps/day) and Non-Active Group (NAG; <10,000 steps/day). Correlations between PA and patient characteristics were studied using the Pearson coefficient. Participants’ user experience was analysed with a questionnaire. The 10,000 steps/day was reached by 57.7% of participants, with 12,603 (1525) and 7495 (1626) being the mean steps/day of the AG and NAG, respectively. In general, no significant variations (*p* > 0.05) in PA levels or adherence to wristband were produced. Only the correlation between very active minutes and arthropathy was significant (*r* = −0.40, *p* = 0.045). Results of the questionnaire showed a high level of satisfaction. In summary, PWH are able to comply with the PA recommendations, and the Fitbit wristband is a valid tool for a continuous and long-term monitoring of PA. However, by itself, the use of a wristband is not enough motivation to increase PA levels.

## 1. Introduction

Haemophilia is a rare inherited haematological disorder that affects the blood coagulation process [1]. The clinical manifestations include an increased tendency for spontaneous bleeding into a joint, called haemarthrosis. Deficient levels of factor VIII (haemophilia A) or factor IX (haemophilia B) affect the severity of bleeding [1,2]. Bleeds in the elbows, knees, and ankles are the most common injuries in haemophilia and often occur during daily activities [3,4]. If repeated episodes are not treated, chronic damage evolves over time into chronic haemophilic arthropathy, which accounts for 75% of complications in haemophilia [4].

In the general population, sedentary lifestyle is associated with cardiovascular disease, cancer, and diabetes [5]. In the case of people with haemophilia (PWH), inactivity may increase due to arthropathy or fear of bleeding, causing overweight and musculoskeletal disorders and, therefore, aggravating the pre-existing arthropathy [6,7,8]. Current therapy regimens with clotting factors minimize the risk of bleeding, allowing PWH to perform physical activity (PA), physical exercise, and sport [9]. Properly managed, PA is highly beneficial for PWH since it improves muscle strength, proprioception, flexibility, and balance, favouring the maintenance of good musculoskeletal health and, therefore, reducing bleeding tendency [6,7,10,11]. Furthermore, in combination with PA, nutrition supplementation (e.g. vitamin D) provide sound physiological rationale for potential additive or synergistic effects that should be beneficial for PWH. [12,13].

For adults aged 18–64 years old, it is recommended to accumulate at least 150 min of moderate-intensity PA throughout the week or an equivalent combination of moderate- and vigorous-intensity activity [14,15]. Another popular recommendation is to accumulate a minimum of 10,000 steps/day [16,17,18]. Based on the number of daily steps, it is possible to classify PA levels using the graduated step index for healthy adults proposed by Tudor-Locke et al.: (1) Sedentary (<5000 steps/day); (2) low-active (5000–7499 steps/day); (3) somewhat active (7500–9999 steps/day); (4) active (10,000–12,499 steps/day); and (5) highly active (≥12,500 steps/day) [16].

In recent years, a large number of consumer PA monitors from companies like Fitbit, Jawbone, Apple, Garmin, Polar, and Xiaomi have appeared on the market. Their popularity has risen in the general public because these devices are affordable, unobtrusive, and user-friendly [19,20]. These devices contain a triaxial accelerometer and, depending on the model, may also contain heart rate sensors, global positioning systems, etc. Using data from these sensors, proprietary algorithms, and user information, these activity monitors can estimate steps, distance travelled, energy consumed, continuous heart rate, levels and types of PA, etc. [19,20,21]. Due to the interest in monitoring PA, these devices (especially the different Fitbit models) have also become very popular in biomedical research [21]. For example, according to searches conducted on clinicaltrials.gov and the PubMed database as of April 2018, Fitbit wristbands have been used in 218 clinical studies and 272 research papers. As reported by Evenson et al., these Fitbit devices have high validity and interdevice reliability in the monitoring of the step count [19].

In the particular case of haemophilia, questionnaires have traditionally been used in the study of PA and participation in sport activities [8,22,23,24,25]. Recently, several studies have employed research-grade accelerometers (ActiGraph GT3X, ActiTrac, etc.) to objectively monitor the amount and intensity of PA [26,27,28,29], but despite being widely used in biomedical research, commercial activity monitors in PWH have been used only in a few works. Using pedometers, Goto et al. reported the number of steps/day in a case report [30] and in a cohort of 16 patients included in a self-monitoring program [31], and Pérez-Alenda et al. quantified the daily PA in seven patients using a Fitbit wristband [32]. Furthermore, the adherence and acceptance of activity trackers has not been studied previously in PWH.

Therefore, the primary goal of the present study was to objectively monitor daily PA and analyse its evolution over time according to the initial PA levels in a cohort of adult patients with haemophilia using a commercial activity tracker. The secondary goals were to analyse the relationship between levels of PA, demographics, and joint health status, as well as to study the acceptance of and adherence to the activity tracker.

## 2. Materials and Methods

### 2.1. Study Design and Participants

The present work is an observational-prospective single-centre study of a group of patients from a local hospital. Inclusion criteria were: (1) Males over 18 years old; (2) diagnosed with severe haemophilia A or B; and (3) under prophylaxis treatment. Exclusion criteria were: (1) Presenting inhibitors (≥5 Bethesda units) and (2) having suffered joint or muscle bleeding in the last three months. During 2015, a total of 125 patients with haemophilia were attended to in routine visits at the hospital, 75 met the inclusion criteria, and finally, 28 agreed to participate in the study. Participation was voluntary and all participants signed a written informed consent. This study conformed to The Declaration of Helsinki and was approved by the Human Research Ethics Committee (H1406715601199) of the University of Valencia. This article adheres to the STROBE guidelines [33].

### 2.2. Procedures

Participants’ data were extracted from medical health records and a personal interview. The following variables were recorded: Age, height, weight, BMI, educational level, computer skills, pharmacological treatment, history of bleeding, and joint health status according to the radiological Pettersson scale (maximum score 78 points, 13 points × 6 joints) [34] and the clinical Haemophilia Joint Health Score 2.1 (HJHS) (maximum score 124 points, 20 points × 6 joints, plus 4 points assigned to global gait) [35]. In both scales, higher values represent a worse outcome. The HJHS evaluation was performed by a senior physiotherapist widely experienced in haemophilic arthropathy.

In addition, on the first visit, a Fitbit Charge HR activity wristband (Fitbit, San Francisco, CA, USA) was delivered to each participant. Although this device records a large number of variables (distance travelled, energy consumed, continuous heart rate, stairs climb, sleep quality, etc.), this study focuses on analyzing the number of steps, active minutes, and wear time. Fitbit wristbands calculate the active minutes using metabolic equivalents (METs) for physical activities (walking, running, cycling, multi-sport, etc.) maintained at least 10 min in a row as follows: <3 METs, light active; 3–6 METs, fairly active; >6 METs, very active. One MET indicates the basal metabolic rate and is based on height, weight, age, and gender. Fitbit uses its own algorithm to obtain the equivalent METs from the number of counts provided by the accelerometer [36].

Participants were informed of the device’s functions and were instructed to wear the wristband continuously from morning to night (except for water-related activities), follow their progress on the Fitbit website or mobile app, and synchronize and charge the wristband at least two times a week to minimize lost data.

During the first week of monitoring, participants were to continue with their usual daily routines. Average daily steps of this first week were considered the steps/day at baseline. After this week, participants were encouraged to try to comply with the recommendation of 10,000 steps/day or to increase the steps/day if they usually reached this recommendation already. However, they were not instructed to perform a new specific physical activity or athletic program. Using the baseline steps/day, participant data were divided into two groups for comparison and analysis: Active Group (AG), formed by participants with a number of steps/day greater than or equal to 10,000, and Non-Active Group (NAG), participants with less than 10,000 steps/day.

After monitoring PA during a 13-week follow-up, participants were called for a second visit to discuss their recorded data with them and to record weight and musculoskeletal bleedings. In all cases, less than 1000 steps/day corresponds to a non-typical day; for example, not having worn the wristband for most of the day. Therefore, a valid day of measurement was defined as a day with more than 1000 steps. In addition, to analyse the experience with the use of the Fitbit wristband, participants completed a technology acceptance questionnaire developed by Mercer et al. [37]. This 17-item questionnaire assesses the domains of external variables, perceived usefulness, perceived ease of use, attitude toward using, behavioural intention to use, and actual system use. Each item can be scored from 1 (strongly disagree) to 5 (strongly agree).

### 2.3. Data Processing and Statistical Analysis

In order to manage and download the data of patients’ wristbands remotely, the researchers developed their own custom-made software. The downloaded data were processed later using Matlab (The MathWorks, Inc., Natick, MA, USA, version R2015a) software.

Normality of the data was verified using the Shapiro–Wilk test. Descriptive measures are shown with the mean and standard deviation. Anthropometric data and questionnaire results from the AG and NAG were compared using an unpaired *t*-test or Wilcoxon rank-sum test, depending on normality. The pre-post weight was compared using a paired *t*-test. Correlations between PA and the patients’ characteristics were analysed using the Pearson coefficient. Results of correlations were interpreted as very weak (*r* < 0.20), weak (*r* ≥ 0.20 and *r* < 0.40), moderate (*r* ≥ 0.40 and *r* < 0.60), strong (*r* ≥ 0.60 and *r* < 0.80), or very strong (*r* ≥ 0.8).

A two-factor analysis of variance (ANOVA) [week range (4) × group (2)] was used to determine differences in physical activity variables over the course of the study period in both groups. The Bonferroni post hoc correction was applied to avoid type I error in the multiple comparisons when the ANOVA analysis indicated significant differences. Statistical significance was considered at *p* < 0.05. All statistical analyses were done in IBM SPSS Statistics for Windows (Version 22.0, IBM Corp., Armonk, NY, USA).

### 2.4. Sample Size

An a priori power analysis was conducted in G* power (Heinrich-Heine-Universität Düsseldorf, Düsseldorf, Germany, version 3.1.9.2) software to calculate the required sample size. With the present study design (ANOVA (4 × 2)), accepting a 5% alpha risk (α = 0.05) as well as a 20% beta risk (β = 0.2; power = 0.8), a total of 24 subjects were required to achieve at least a medium effect size (*f* = 0.25; *d* = 0.5).

Regarding the correlation analysis, with α = 0.05, power = 0.8, and the 26 final participants, the minimum effect size detectable is *r* = 0.52.

## 3. Results

### 3.1. Participants

The group of 75 patients who met the inclusion criteria had a mean age of 37.24 (11.07) years old and presented a mean Pettersson score of 32.05 (21.34) points, while the study group had a mean age of 36.08 (9.54) years old and a mean Pettersson score of 29.40 (21.17) points. Since the between-group differences were not statistically significant (*p* > 0.05), the study group was representative with respect to the total sample in age and degree of arthropathy.

Two of the patients dropped out of the study due to personal reasons, with 26 participants constituting the final sample. All participants had computer skills and habitually used smartphones. According to the baseline steps/day, the AG and NAG were composed of 15 and 11 participants, respectively. Participant characteristics are shown in Table 1. The between-group analysis did not show significant differences at baseline.

After the 13-week follow-up, the AG presented a weight of 76.85 (15.33) kg and the NAG a weight of 79.46 (27.10) kg, with no significant differences with respect to baseline values (*p* > 0.05). During the monitoring period, four patients suffered bleeds in load joints (three patients from AG and one patient from NAG). The bleeds in the AG were all minor and not related to PA: One ankle haemarthrosis, provoked by ankle sprain while working the same day the patient received the prophylactic treatment; one spontaneous hip haemarthrosis, one day after the last FVIII infusion (mean steps/day: 13,220; steps on the day of bleeding: 12,925); and spontaneous bleeding in the knee in a patient who received daily prophylactic treatment (mean steps/day: 12,347; steps on the day of bleeding: 12,249 steps). In the NAG, one patient suffered two bleeds: One minor spontaneous bleed in the knee, 48 h post infusion of FVIII (mean steps/day: 7200; steps on the day of bleeding: 8176), and one ankle haemarthrosis related to PA caused by a long walk in a short time, 24 h after the last FVIII administration (steps on the day of bleeding: 12,407).

### 3.2. Physical Activity Monitoring

**Figure 1:**
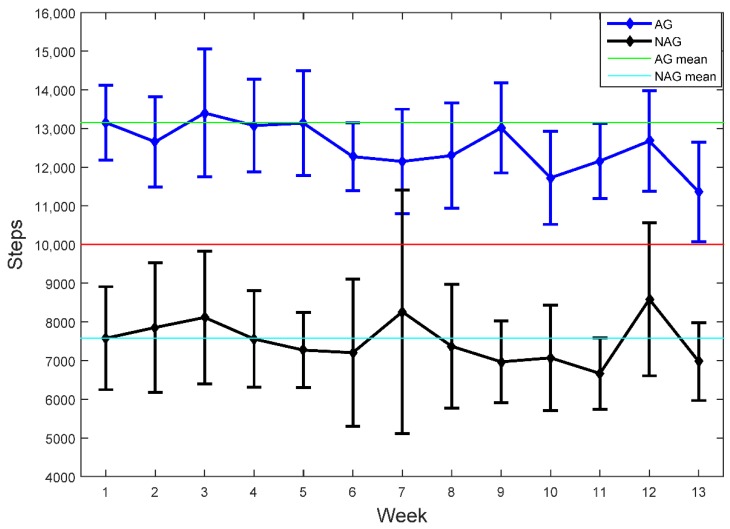
Daily mean steps (and 95% confidence interval) per week for AG (Active Group) and NAG (Non-Active Group). Red line indicates the recommendation of 10,000 steps/day. Green line and cyan line indicates the daily mean steps at baseline for the AG and NAG, respectively.

According to the Tudor-Locke et al. scale at baseline, one of the participants was sedentary, five were low-active, five were somewhat active, six were active, and nine were highly active, with 57.7% of the participants reaching the target of 10,000 daily steps. The average of the 26 participants was 10,441 (2997) steps/day (12,603 (1525) steps/day in the AG and 7495 (1626) steps/day in the NAG). For the AG, the mean number of steps/day in week one was 13,152 (1748) and remained similar in weeks 2–5 and 6–9, but decreased significantly (*p* = 0.027) in weeks 10–13. Regarding the NAG, the mean number of steps/day was 7578 (1979) at baseline, with no significant differences (*p* > 0.05) compared with the other periods. In both groups, the number of active minutes (light, fairly, and very active) remained constant over the weeks, with no significant differences (Table 2). Throughout the study period, the 84.6% of participants reached the 150 min of PA per week. Figure 1 shows the daily mean steps (and 95% confidence interval) per week for both groups.

The between-group analysis indicates that the AG presented higher values than the NAG in the number of steps (*p* < 0.001), distance (*p* < 0.001), and levels of activity (light, fairly, and very active min/day) (*p* < 0.05). These results were repeated in all periods analysed except for the fairly active minutes in weeks 10–13 (*p* > 0.05).

Of the 91 total recorded days, the mean valid days (>1000 steps/day) was 86.8 (6.5). At baseline, the average wear time (min/day) was 912.3 (107.6) for the AG and 870.9 (105.3) for the NAG, remaining high throughout the study period and comparable across groups and weeks with no significant differences (*p* > 0.05) (Table 2).

### 3.3. Correlation Analysis

In general, the number of steps and minutes of activity (light, fairly, and very active) do not show significant correlations with age, BMI, and HJHS (Table 3). Only the correlation between very active minutes and HJHS score was significant (*p* = 0.045), but this correlation was weak (*r* = −0.40).

### 3.4. Technology Acceptance Questionnaire

Results are shown in Table 4. The total average was high (4.24 (0.51) for AG and 4.19 (0.42) for NAG), with no significant differences between groups. The items with the highest scores were: “I found it easy to learn to operate the activity tracker” and “Overall, the activity tracker was easy to use”.

## 4. Discussion

In this work, a commercial activity tracker was used to perform a 13-week monitoring of daily PA in 26 adults with haemophilia. Our results showed that 57.7% of participants achieved the goal of 10,000 daily steps, and the 84.6% reported a mean number of active minutes greater than the recommended minimum to be healthy.

PA levels of the general population depend on multiple factors, such as country, sex, age, race/ethnicity, etc. [38,39]. Tudor-Locke et al. concluded that healthy adults typically perform a number of daily steps between 4000 and 18,000 [38]. For example, in male adults, Bassett et al. reported an average of 5340 (3509) steps/day in a U.S. sample (*n* = 526), and Cocker et al. reported 9906 (5046) steps/day in a Belgian population (*n* = 598) [40,41]. In PWH, Goto et al. showed a maximum of 5000 steps/day in a single patient and an average of 5805 (3384) in a sample of 16 patients (self-monitoring group), and research by Pérez-Alenda et al. showed a median of 10,358 steps/day in a sample of seven participants [30,31,32]. Comparatively, the number of steps taken by the AG in our study is higher than those reported by Pérez-Alenda et al. and Goto et al., and even superior to the results of some studies conducted in the general population. Moreover, the mean number of steps of the NAG is higher than that showed by Goto et al. and Bassett et al., but lower than the rest of the studies analysed. In addition, our results showed that both groups comply with the recommendations of active minutes per week [14,15]. However, it should be noted that the exact algorithms used by Fitbit to classify PA levels using sensor data are unknown to consumers and researchers due to proprietary concerns, making it difficult to compare PA levels between the devices of different companies [21,36].

In addition to factors involved in the general population, levels of PA in PWH may decrease depending on age, BMI, and arthropathy [8,42]. In our study, the NAG presented a higher level of arthropathy than the AG, but this difference was not significant (*p* > 0.05). Furthermore, no strong correlations were found between the analysed variables. Only the relationship between very active minutes and joint health status was significant (*p* = 0.045), but this correlation was weak (*r* = −0.40).

Many of the studies performed with research accelerometers are carried out over a short period of seven-day monitoring [16]. This is due to the storage capacity and the need to return devices to researchers to extract the data. However, the data recorded with consumer trackers can be frequently transferred to a website [21]. Another important feature of these devices is that, according to some studies, they present good accuracy, validity, and reliability in the step count, and therefore, they are suitable for research purposes [19,20,43]. These characteristics have allowed us to obtain objective data remotely and to be able to analyse the evolution of PA levels over a long period. Our results show a high number of valid recorded days and minutes of wear time, allowing us to ensure that results are not biased by the amount of time the wristband was used. In addition, the wear time was maintained throughout the study period with no significant decline, demonstrating a very good adherence. Given the PA levels at baseline, no significant variations in the number of steps and active minutes were produced during the weeks monitored, with the exception of the steps/day in the AG. In this case, a significant decrease (*p* = 0.027) of the steps/day was observed in weeks 10–13.

Regarding the usability and usefulness of the Fitbit Charge HR, both groups presented high scores on the technology acceptance questionnaire, indicating that participants were satisfied with the fitness tracker. These scores are higher than those obtained in the devices analysed in the work of Mercer et al. [37]. Given these results, the Fitbit Charge HR has been shown to be an adequate device for continuous and long-term monitoring of PA, but nevertheless, their use does not seem to be sufficient motivation to increase levels of PA in PWH, it being necessary to complement the use of the wristband with additional motivational strategies [30].

Finally, during the study period, three patients in the AG suffered minor bleeds not related to PA in load joints. In the NAG, one patient registered two bleeds, one of them due to a considerably higher than usual level of PA. Hence, despite arthropathy, PWH in prophylactic treatment are able to comply with PA recommendations for health with a minimal risk of bleeding.

Our study sample was limited to patients attended to in a single haemophilia centre, and, therefore, not all results can be extrapolated to the general haemophilia population. Furthermore, future studies are needed to investigate the use of the activity tracker in combination with additional strategies to encourage PA.

## 5. Conclusions

The results indicate that, despite arthropathy and thanks to the prophylactic treatment, adult patients with haemophilia are able to comply with PA recommendations with a minimal risk of bleeding. In addition, commercial activity trackers are suitable for continuous and long-term monitoring of PA in PWH, due to their technological characteristics as well as the high degree of wear adherence and satisfaction of use. Therefore, this type of device can help healthcare providers to optimize outcomes and make better use of available resources, allowing a tailored prophylaxis therapy based on the objective PA level of their patients. However, in addition to the wristband, it seems necessary to use additional motivational strategies to increase PA levels in non-active patients.

## Figures and Tables

**Table 1:** Characteristics of the study participants at baseline.

Demographics	All (*n* = 26)	AG (*n* = 15)	NAG (*n* = 11)	*p* Value
Characteristics, mean (SD)				
Age (years)	36.08 (9.54)	35.60 (9.77)	36.73 (9.65)	0.77
Height (m)	1.74 (0.07)	1.75 (0.07)	1.73 (0.07)	0.37
Weight (kg)	77.70 (19.80)	76.76 (14.89)	78.98 (25.81)	0.64
BMI (kg/m^2^)	25.53 (5.52)	25.00 (4.37)	26.24 (6.97)	0.88
HJHS total score	27.92 (16.61)	24.73 (9.77)	32.27 (9.65)	0.26
Pettersson total score	29.40 (21.17)	24.67 (19.98)	36.50 (21.92)	0.17
Prophylaxis weekly dose (IU/kg)	27.80 (10.37)	26.61 (10.07)	29.42 (11.04)	0.53
Prophylaxis dosing regimen, *n* (%)				
Two days a week	11 (42.31)	4 (26.67)	7 (63.64)	-
Three days a week	14 (53.85)	10 (66.67)	4 (36.36)	-
Daily	1 (3.85)	1 (6.67)	0	-
Education level, *n* (%)				
Less than high school	1 (3.85)	1 (6.67)	0 (0.00)	-
High school graduate	3 (11.54)	1 (6.67)	2 (18.18)	-
Some college	10 (38.46)	6 (40.00)	4 (36.36)	-
College graduate	12 (46.15)	7 (46.67)	5 (45.45)	-

AG: Active Group; NAG: Non-Active Group; BMI: Body Mass Index; IU: International Units. HJHS: Haemophilia Joint Health Score.

**Table 2:** Physical activity data registered by Fitbit Charge HR during the 13-week follow-up.

Variable	Week 1 (Baseline)	Weeks 2–5	Weeks 6–9	Weeks 10–13	*p*1	*p*2	*p*3
	Active Group (*n* = 15)						
Steps/day	13,151.7 (1748.2)	13,067.2 (2407.5)	12,434.1 (2146.0)	11,979.9 (2164.2)	1	0.99	0.027 ^ǂ^
Light active (min/day)	294.3 (67.6)	282.9 (68.1)	282.8 (62.9)	270.6 (72.2)	0.97	1.00	0.23
Fairly active (min/day)	39.2 (33.1)	44.6 (34.5)	45.8 (34.9)	48.8 (52.9)	0.64	0.39	0.83
Very active (min/day)	40.2 (24.4)	38.1 (25.3)	33.8 (19.4)	31.5 (23.5)	1	0.28	0.05
Wear Time (min/day)	912.3 (107.6)	873.4 (146.0)	875.4 (134.6)	870.7 (165.4)	0.26	0.28	1
	Non-Active Group (*n* = 11)						
Steps/day	7577.5 (1979.4) **	7701.1 (2087.0) **	7462.6 (3033.6) **	7324.8 (2121.7) **	1	1	1
Light active (min/day)	220.7 (57.4) *	219.2 (48.9) *	207.1 (57.7) *	200.0 (44.6) *	1	1	0.68
Fairly active (min/day)	15.8 (9.5) *	16.2 (14.1) *	18.9 (19.1) *	17.3 (13.6)	1	1	1
Very active (min/day)	15.5 (11.6) *	15.8 (15.7) *	14.2 (15.8) *	15.0 (13.0) *	1	1	1
Wear Time (min/day)	870.9 (105.3)	851.6 (147.7)	848.0 (155.3)	857.8 (158.1)	1	1	1

Values are mean (standard deviation). *p*1: Within-group differences at baseline and weeks 2–5; *p*2: Differences at baseline and weeks 6–9; *p*3: Differences at baseline and weeks 10–13. ^ǂ^: Indicate significant differences. *: Between-group significant differences (*p* < 0.05), **: Between-group significant differences (*p* < 0.001).

**Table 3:** Results of correlation analysis.

Demographics	Steps		Light Active		Fairly Active		Very Active	
	*r*	*p*	*r*	*p*	*r*	*p*	*r*	*p*
Age (years)	−0.11	0.61	0.14	0.51	0.17	0.39	−0.37	0.07
BMI (kg/m^2^)	−0.18	0.30	−0.18	0.37	0.07	0.75	−0.02	0.90
HJHS score	−0.25	0.22	−0.13	0.52	−0.13	0.53	−0.40	**0.045**

BMI: Body Mass Index; *r*: Pearson correlation coefficient. *p*: *P* significance value. Statistically significant values are showed in bold.

**Table 4:** Participant experience questionnaire.

Item	AG		NAG		*p*
Overall, I was satisfied with the activity tracker.	4.40	(0.63)	4.45	(0.69)	0.79
Using the activity tracker helped me set activity goals.	4.07	(0.88)	4.09	(0.54)	0.86
Using the activity tracker helped me reach my activity goals more rapidly.	3.93	(0.96)	3.91	(0.54)	0.84
Using the activity tracker helped me to be more active.	4.36	(0.74)	3.91	(0.70)	0.13
Using the activity tracker made it easier to be more active.	4.00	(1.00)	4.09	(0.54)	1
Using the activity tracker supported me in managing my disease.	2.93	(0.59)	3.00	(1.10)	0.86
I found it easy to learn to operate the activity tracker.	4.73	(0.59)	4.55	(0.69)	0.40
I found the activity tracker to be clear and understandable to use.	4.60	(0.63)	4.45	(0.69)	0.57
I found the activity tracker to be flexible to work with.	4.60	(0.63)	4.36	(0.67)	0.33
Overall, the activity tracker was easy to use.	4.73	(0.46)	4.55	(0.52)	0.35
People who influence my behaviour would think I should use the activity tracker.	3.33	(1.23)	3.73	(0.90)	0.36
People who are important to me would think I should use the activity tracker.	3.47	(1.19)	3.73	(0.90)	0.55
I have the technology necessary to use the activity tracker.	4.67	(0.49)	4.73	(0.47)	0.77
I have the knowledge necessary to use the activity tracker.	4.67	(0.49)	4.73	(0.47)	0.77
The activity tracker was compatible with other systems I use.	4.47	(0.74)	4.36	(0.67)	0.62
I am very knowledgeable about my physical activity needs.	4.47	(0.64)	4.45	(0.69)	1
I understand how to use physical activity to manage my health problems.	4.40	(0.74)	4.09	(0.83)	0.34
The activity tracker was comfortable to wear.	4.47	(0.92)	4.36	(0.67)	0.44
The activity tracker accurately tracked my physical activity.	4.20	(1.01)	4.00	(0.63)	0.36
Total	4.24	(0.51)	4.19	(0.42)	0.37
